# Broadening the aims of avian influenza surveillance according to the One Health approach

**DOI:** 10.1128/mbio.02111-24

**Published:** 2024-09-18

**Authors:** Thijs Kuiken

**Affiliations:** 1 Department of Viroscience, Erasmus University Medical Centre, Rotterdam, the Netherlands; University of Florida College of Public Health and Health Professions, Gainesville, Florida, USA

**Keywords:** influenza, surveillance studies, One Health, wildlife, infectious disease

## Abstract

The ongoing outbreak of the Goose/Guangdong/1/1996 (Gs/Gd) H5 lineage of highly pathogenic avian influenza (HPAI) viruses has caused higher mortality than all other HPAI outbreaks taken together. It is unique in its spillover and adaptation to wild waterfowl, which has facilitated its spread worldwide to many other species. However, avian influenza virus (AIV) surveillance, which historically aims to protect the poultry sector, is inadequate to document the spread and impact of Gs/Gd H5 virus in wild birds and other wildlife in most countries. A positive exception is Canada’s AIV surveillance in wild birds, applied in a recent study (J. A. Giacinti, A. V. Signore, M. E. B. Jones, L. Bourque, et al., mBio 15:e03203-23, 2024, https://doi.org/10.1128/mbio.03203-23), which aims to protect wildlife, domestic animals, and human health according to the One Health approach. It is recommended to follow this approach in other countries to fill knowledge gaps in the epidemiology of Gs/Gd H5 virus in wild birds and other wildlife and to help control and, above all, prevent future HPAI outbreaks.

## COMMENTARY

When a new variant of the highly pathogenic avian influenza (HPAI) virus was diagnosed on a commercial poultry farm in 1996, sickening 40% of its domestic geese (1), who would have thought it would lead to the biggest HPAI outbreak in history? Yet, that virus was the ancestor of the so-called Goose/Guangdong/1/1996 (Gs/Gd) subtype H5 lineage of HPAI viruses, which has caused the deaths of over 400 million chickens and other poultry, more than those caused by all the other 36 HPAI outbreaks since 1959 taken together (2). It also has caused severe human illness, mainly through contact with infected poultry, resulting in the deaths of 457 people out of 868 with confirmed Gs/Gd H5N1 virus infection (3).

Like other HPAI viruses, the genesis of the Gs/Gd H5 virus was likely due to mutation from a low pathogenic avian influenza virus that had somehow entered the poultry farm (4). However, in contrast to other HPAI viruses, which are typically restricted to poultry, the Gs/Gd H5 virus spilled over into wild waterfowl and spread worldwide (5, 6). This spillover was facilitated by the failure to eliminate the virus from poultry populations in Asia, and the custom to graze poultry (mainly domestic ducks and geese) in wetlands, which provided ample opportunity for virus transmission to wild waterfowl (7).

Wild waterfowl became both a vector and a victim of the Gs/Gd H5 virus: they transported the virus over long distances during migration (5), yet also suffered high mortality (8, 9). The virus spread to other free-living wildlife and to farmed animals in countries at great distance from Asia. Infected farm animals include cattle in the United States, where—as of 12 August 2024—Gs/Gd H5 virus has been confirmed in dairy cows of 189 herds in 13 states (https://www.aphis.usda.gov/livestock-poultry-disease/avian/avian-influenza/hpai-detections/hpai-confirmed-cases-livestock) and in four people working on dairy farms where cows tested positive (https://www.cdc.gov/media/releases/2024/p-0703-4th-human-case-h5.html). Based on the spillover of this virus from dairy cows to people, the future pandemic potential risk from Gs/Gd H5 was classified as “moderate” (https://www.cdc.gov/bird-flu/spotlights/ah5n1-response-update.html). Worldwide, the Gs/Gd H5 virus is present in Asia, Europe, Africa, North America, South America, and Antarctica and has been reported in 356 species of wild birds (10) and 58 species of non-human mammals (11). There are several documented cases of these Gs/Gd H5 infections causing unparalleled high mortality in wild bird and mammal populations, e.g., references 12, 13.

Although current avian influenza virus (AIV) surveillance in wild birds—which often consists of testing a limited number of wild birds found dead—may be sufficient as early warning for the poultry sector, it does not provide adequate information to understand the epidemiology and impact of Gs/Gd H5 virus in wild bird populations. Furthermore, monitoring, reporting, and responding to die-offs of wild birds from HPAI in most countries are poorly planned and poorly funded. As a consequence, current AIV surveillance in wild birds shows large geographical gaps and does not provide an estimate of the immediate and long-term impacts of the Gs/Gd H5 outbreak on wild bird populations (14). This is illustrated by the information available in the database of the World Animal Health Information System. Despite the high mortality of wild birds from Gs/Gd H5 virus infections worldwide, only 75,106 reports of Gs/Gd H5 virus infection have been recorded between 2016 and 2023, and case data reporting is highly inconsistent. It may include all observed carcasses, only those tested and confirmed, or no information on numbers at all. As a result, the available wild bird data are deficient in providing an understanding of the scale of these wild bird mortalities (14).

Therefore, AIV surveillance requires a broader view, such as the One Health approach. The aim of the One Health approach, as endorsed by WHO, FAO, WOAH, and UNEP, is to “sustainably balance and optimize the health of people, animals, and ecosystems” (15). The implication of this approach is not only that multiple sectors (e.g., public health, agriculture, nature conservation) work together to foster well-being and tackle threats to health and ecosystems but also that the goals of their activities are in line with the One Health aim.

The recent article by Giacinti and colleagues (16) is a good example of the application of this One Health approach to AIV surveillance in wild birds. The stated aim of Canada’s Interagency Program for Avian Influenza Viruses in Wild Birds, through which the authors performed this study, is to “understand, evaluate, and mitigate the risk of HPAI viruses to wildlife, domestic animal, and human health.” The authors characterized the spread of Gs/Gd H5 virus across Canada in the first year after its incursion into the country. They found that 27.4% out of 6,246 sick and dead wild birds tested were positive for HPAI virus across 80 species and that 5.2% out of 11,295 apparently healthy wild birds tested were positive for HPAI virus across 19 species. Notable mortality events associated with HPAI virus infection were reported in over 20 species of raptors, waterfowl, seabirds, and other wild birds. They concluded that no other infectious disease has caused this magnitude of mortality in so many wild bird species in Canada and that the combination of increased mortality and decreased reproduction from Gs/Gd H5 virus infection can result in significant population-level impacts, particularly for those species or populations that are vulnerable or already experiencing multiple concurrent anthropogenic stressors.

Canada’s AIV surveillance program in wild birds is an example for other countries. It is in line with FAO’s Global Consultation on Avian Influenza (14), where it was recommended to recognize HPAI not only as a concern for poultry production and public health but also as a concern for wildlife conservation; and to change goals, methods, and implementation of AIV surveillance, research, and response in wild birds accordingly at national, regional, and global levels. It was further recommended to make international funds, laboratory support, and capacity building available to low- and middle-income countries to improve geographical and species coverage of AIV surveillance in wild birds sustainably at the global level.

A key aspect of infectious disease surveillance is the cycle of detecting, responding to, and—last but not least—preventing outbreaks (17). In the case of HPAI in wildlife, there are few if any response options to mitigate the impact of the disease once it has spilled over from poultry. Therefore, it is all the more important to prevent the upstream events that favor the genesis and spread of HPAI viruses on poultry farms (18, 19), as well as the spillover of HPAI from poultry to wild waterbirds in wetland areas (7). Prevention is better than cure.
